# Comparative Transcriptomic Analysis Reveals the Potential Molecular Mechanism Underlying Squalene Biosynthesis in Developing Seeds of Oil-Tea (*Camellia oleifera*)

**DOI:** 10.3390/ijms26125465

**Published:** 2025-06-07

**Authors:** Xu Gu, Anmin Yu, Ping Li, Meihong Zhang, Ya Lv, Debing Xu, Aizhong Liu

**Affiliations:** 1 College of Forestry, Southwest Forestry University, Kunming 650224, China; guxu675129144@163.com (X.G.); anminyu@swfu.edu.cn (A.Y.); liping2020@swfu.edu.cn (P.L.); zhangmh_0724@swfu.edu.cn (M.Z.); lvya2017@163.com (Y.L.); 2 Key Laboratory for Forest Resources Conservation and Utilization in the Southwest Mountains of China, Ministry of Education, Southwest Forestry University, Kunming 650224, China; 3 Yunnan Academy of Forestry and Grassland, Kunming 650201, China; xudebing@yafg.ac.cn

**Keywords:** seed development, squalene accumulation, comparative transcriptomics, transcriptional regulation, MYC2

## Abstract

Oil-tea (*Camellia oleifera*), a typical oilseed tree, produces high-quality edible vegetable oils that contain rich unsaturated fatty acids and diverse lipid-soluble active compounds such as squalene. Although squalene biosynthesis and its molecular regulation have been studied in several plants, the molecular mechanisms underlying squalene biosynthesis in oil-tea seeds remain uncertain. We investigated and determined squalene accumulation with seed development. We conducted comparative transcriptomic analyses using the RNA-seq technique at the early, fast biosynthesis, and late stages of squalene accumulation with oil-tea seed development and identified 13 squalene biosynthesis key enzyme genes (such as *CoHMGR_4*, *CoAACT_2*, *CoFPS_1*, and *CoFPS_2*) in developing oil-tea seeds. According to whether the expressions of key enzyme genes were associated with squalene accumulation we found that the precursor IPP of squalene biosynthesis obtained via the MVA pathway was dominant with oil-tea seed development. Based on the gene co-expression analyses, we identified multiple transcription factors potentially involved in regulating squalene biosynthesis such as *CoMYC2*, *CoREM39*, *CobZIP5*, *CoERF* and *CoWRKY*. Using yeast one-hybrid and dual-luciferase assay experiments we demonstrated that the transcription factor *CoMYC2* could activate the expression of a key enzyme gene *CoHMGR_4*, suggesting that *CoMYC2* might be a critical regulator during squalene biosynthesis in oil-tea seed development. This study gives not only insights into understanding the molecular basis of squalene biosynthesis in oil-tea developing seeds but also provides gene resources for developing genetically improved varieties with higher content of squalene in oil-tea.

## 1. Introduction

Oil-tea (*Camellia oleifera* Abel.) is a typical oilseed tree that is widely planted in many regions of Southern China and other Asian countries. The planting area of oil-tea reached 4.5 million hectares in China in 2022. The oil content of oil-tea seed is often relatively high though it is variable among different varieties [1,2,3]. Its seed oil (Camellia oil) has been considered a high-qualified vegetable oil because it is composed of mainly unsaturated fatty acids (FAs) such as oleic acid (C18:1), linoleic acid (C18:2), and palmitic acid (C16:0) [4,5]. Notably, Camellia oil contains diverse lipid-soluble compounds such as squalene (SQ), vitamin E, and sterols, which are considered to be critical in participating in various physiological metabolisms for maintaining human health [1,6]. Thus, Camellia seed oil has been used as a high-value edible vegetable oil in China for more than two thousand years, and it is believed to be physiologically beneficial for preventing and treating cardiovascular and cerebrovascular diseases by reducing the level of blood cholesterol and thrombosis [7,8]. In particular, the rich lipid-soluble compound squalene, a polyunsaturated hydrocarbon terpenoid (C30H50) in Camellia oil, has been extensively applied as an active compound in the pharmaceutical, nutraceutical, and cosmetic industries because of its functions as an antioxidant and detoxifying agent [9,10,11]. Due to its commercial importance, the demand for squalene has been growing in recent years.

Squalene often accumulates with storage lipid biosynthesis in developing seeds or oleaginous tissues, such as fruits of olive, seeds of camellia, and amaranth [1,12,13]. Generally, biosynthesis of squalene starts from the precursor isopentenyl diphosphate (IPP), which is derived via two independent pathways: the cytosolic-localized mevalonate (MVA) pathway and the plastid-localized methylerythritol phosphate (MEP) pathway [11,14]. Substrate acetyl-CoA, within the MVA pathway, is used to synthesize IPP via the catalyzing of acetyl-CoA acetyltransferase (AACT), 3-hydroxy-3-methylglutaryl-CoA synthase (HMGS), 3-hydroxy-3-methylglutaryl-CoA reductase (HMGR), mevalonate kinase (MVK), phosphomevalonate kinase (PMK), and mevalonate diphosphate decarboxylase (MVD). Then, isopentenyl diphosphate isomerase (IDI) catalyzes IPP to the isomerization of dimethylallyl diphosphate (DMAPP). The MEP pathway, which can simultaneously synthesize IPP and DMAPP, begins with pyruvate and D-glyceraldehyde-3-phosphate (G3P) and is catalyzed by 1-deoxy-D-xylulose-5-phosphate synthase (DXS), 1-deoxy-D-xylulose-5-phosphate reductoisomerase (DXR), 2-*C*-methyl-D-erythritol 4-phosphate cytidyltransferase (MCT), 4-(cytidine 5′-diphospho)-2-*C*-methyl-D-erythritol kinase (CMK) and 2-*C*-methyl-D-erythritol 2,4-cyclodiphosphate synthase (MDS), 4-hydroxy-3-methylbut-2-enyl-diphosphate synthase (HDS), and 4-hydroxy-3-methylbut-2-enyl diphosphate reductase (HDR). Two molecules of IPP and one molecule of DMAPP are assembled into farnesyl diphosphate (FPP) by farnesyl diphosphate synthase (FPS). Then, two molecules of FPP are condensed into squalene by squalene synthase (SS). Studies have found that key enzyme genes that restrict squalene biosynthesis (including its downstream metabolites) vary in different species. In *Medicago sativa*, squalene production was significantly enhanced when the key enzyme gene *MsAACT1* was overexpressed [15]. In *Arabidopsis thaliana*, overexpression of the key enzyme gene *AtSS* [16] or loss of *AtHMGR* [17] can significantly increase or reduce triterpenoid production, respectively. The overexpression of *PgMVD* and *PgFPS* was found to enhance the accumulation of phytosterols and triterpenoid saponin in *Panax ginseng* hairy roots [18]. Moreover, studies have found that some genes involved in squalene biosynthesis (such as *HMGR*, *FPS,* and *SS*) were regulated by various transcription factors (TFs). In *Hevea brasiliensis*, *HbFPS1* was positively regulated by *HbWRKY27* [19]. In *P. notoginseng*, *PnSS* was positively regulated by PnERF1 and overexpressed *PnERF1* promoted triterpenoid saponin biosynthesis [20]. In the overexpressed transformants of *BpMYB21*, the expression levels of *HMGR*, *FPS*, and *SS* were significantly up-regulated in *Betula platyphylla*, resulting in an increased squalene content [21]. In *M. truncatula*, *MtHMGR1* was regulated by TFs *TSAR1* and *TSAR2*, and the overexpressed transformants of *TSAR1* and *TSAR2* significantly enhanced triterpene saponin accumulation [22]. Therefore, key genes involved in squalene biosynthesis are regulated by diverse TFs in different plants. For oil-tea, the molecular mechanisms underlying squalene biosynthesis remain largely unknown.

The available genome data of oil-tea provides a great opportunity to identify the key genes and regulators involved in squalene biosynthesis and accumulation with seed development. In this study, we first investigated the trends of squalene accumulation with oil-tea seed development; secondly, we identified candidate genes and regulators involved in squalene biosynthesis based on comparative transcriptomic analyses; further, we confirmed that a key enzyme gene involved in squalene biosynthesis is regulated by a transcription factor by experimental tests. This work gives not only insights into understanding the molecular basis of squalene biosynthesis in developing seeds of oil-tea but also provides gene resources for developing genetically improved varieties with a higher content of squalene in oil-tea.

## 2. Results

### 2.1. Determination of Squalene Accumulation with Seed Development

**Figure 1:**
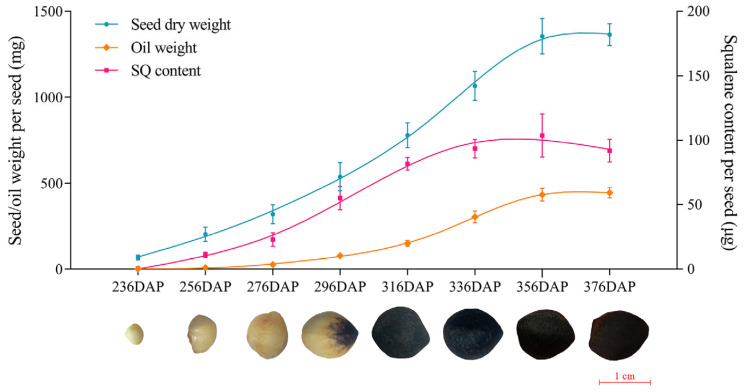
Changes in the seed phenotype, oil, and squalene accumulation with seed development (the mean ± SD). The mean and standard deviation values were calculated based on three biological replicates. Weight per seed (mg) means the seed dry weight (mg) and oil weight (mg) of a single seed. Content per seed (μg) means the squalene content (μg) of a single seed.

The seed development of oil-tea usually lasts approximately 13 months from October to November of the following year. To investigate how squalene is accumulated with seed development, we inspected the content of squalene at different stages of seed development, starting from the early stage 236 DAPs (Days After Pollination) to the mature stage 376 DAPs, covering eight sampling time points, as shown in Figure 1. The dry weight and oil weight (single seed) increased from 67.28 ± 15.22 mg and 1.85 ± 0.40 mg at 236 DAPs to 779.81 ± 72.53 mg and 149.56 ± 16.83 mg at 316 DAPs, reached 1355.54 ± 103.48 mg and 433.09 ± 36.61 mg at 356 DAPs, and up to 1364.59 ± 64.35 mg and 444.56 ± 30.90 mg at 376 DAPs, respectively. The accumulation of squalene was low, approximately 0.45 ± 0.12 μg at 236 DAPs, and rapidly increased to 81.67 ± 4.92 μg at 316 DAPs, slowly climbed to 103.64 ± 16.80 μg at 356 DAPs, and reached 91.94 ± 8.84 μg at 376 DAPs. The analysis of squalene content changes revealed that squalene accumulation does not occur at the initial stage of seed development (up to 236 DAPs). The rapid squalene accumulation stage occur the middle stage of seed development (from about 240 to 336 DAPs), whereas its accumulation slows down at the late stage of seed development (after 336 DAPs). Our results indicated that squalene fast biosynthesis mainly occurred in the middle stage of seed development, and its accumulation trend was consistent with the oil accumulation.

### 2.2. Comparative Transcriptomic Analysis of Developing Seeds

**Figure 2:**
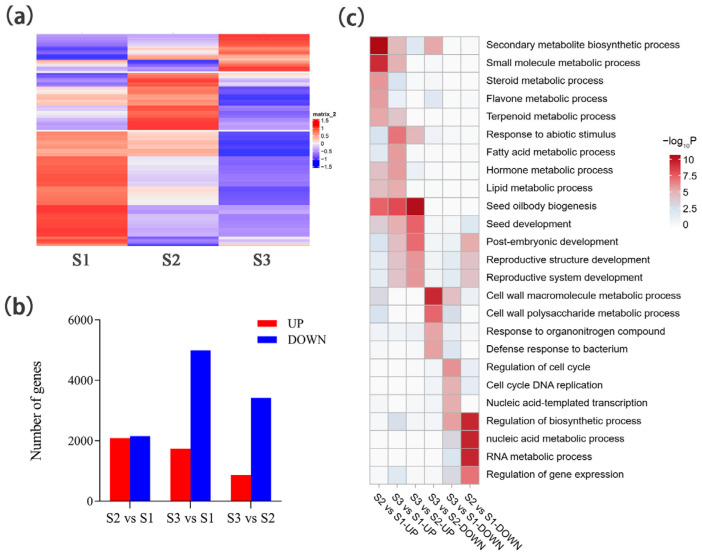
Comparative transcriptomic analysis of genes with RPKM > 1 in three developmental stages of oil-tea seeds. (**a**) The gene expression pattern of three developmental stages. (**b**) Numbers of DEGs. (**c**) GO enrichment analysis of DEGs. S2 vs. S1 indicates the DEGs of the S2 stage compared to the S1 stage. S3 vs. S1 indicates the DEGs of the S3 stage compared to the S1 stage. S3 vs. S2 indicates the DEGs of the S3 stage compared to the S2 stage. UP indicates up-regulated genes. DOWN indicates down-regulated genes.

To identify the key genes involved in squalene biosynthesis in developing seeds of oil-tea, comparative transcriptomic analyses were performed using the high throughput RNA-seq technique. The developing seeds were sampled at 236 DAPs (S1 stage), 256 DAPs (S2 stage), and 316 DAPs (S3 stage), reflecting the early, fast biosynthesis, and late stages of squalene accumulation, respectively. In total, we generated 61.62 Gb of clean data after filtering raw data for further analyses, ranging from 6.46 to 7.12 Gb for each sample, as shown in Table S1. There were 85.45 to 86.99% of clean reads that could be mapped to the oil-tea reference genome (Table S1). The Pearson correlation coefficients ranged from 0.80 to 1.00 among three samples of each stage, indicating the transcriptomic data were good among three replicates (Figure S1). In total, 16,438 genes expressed in at least one stage were identified (with fragments per Kilobase Million (RPKM) > 1). At the S1 stage, 8882 genes were highly expressed (Figure 2a), which were functionally involved in cell growth and development such as DNA replication (ko03030) and homologous recombination (ko03440) (Figure S2a). At the S2 stage, 4996 genes were highly expressed (Figure 2a), which were functionally involved in diverse metabolite pathways such as carbon metabolism (ko01200), cysteine and methionine metabolism (ko00270), and steroid biosynthesis (ko00100) (Figure S2b). At the S3 stage, 2560 genes were highly expressed (Figure 2a), which were functionally involved in metabolism pathways such as carbon metabolism (ko01200), fatty acid metabolism (ko01212), glycerolipid metabolism (ko00561), and alpha-linolenic acid metabolism (ko00592) (Figure S2c).

Based on differential expressions of the 16,438 identified genes among S1, S2, and S3 stages, 8576 differentially expressed genes (DEGs) were identified. Compared with the S1 stage, 4239 DEGs were identified with 2088 up-regulated and 2151 down-regulated genes at the S2 stage (Figure 2b). These up-regulated DEGs were functionally involved in diverse secondary metabolic processes, such as the steroid metabolic process (GO:0008202), flavone metabolic process (GO:0051552), and terpenoid metabolic process (GO:0006721) (Figure 2c). The down-regulated DEGs were functionally involved in the gene expression process, such as the nucleic acid metabolic process (GO:0090304), RNA metabolic process (GO:0016070), and regulation of gene expression (GO:0010468) (Figure 2c). Compared with the S1 stage, 6728 DEGs were identified with 1738 up-regulated and 4990 down-regulated genes at the S3 stage (Figure 2b). These up-regulated DEGs were functionally involved in hormone and lipid metabolic processes such as the hormone metabolic process (GO:0042445), fatty acid metabolic process (GO:0006631), and lipid metabolic process (GO:0006629) (Figure 2c). The down-regulated DEGs were functionally involved in cell cycle processes such as regulation of the cell cycle (GO:0051726) and cell cycle DNA replication (GO:0044786) (Figure 2c). Compared with the S2 stage, 4283 DEGs were identified with 866 up-regulated and 3417 down-regulated genes at the S3 stage (Figure 2b). These up-regulated DEGs were functionally involved in lipid biosynthesis and seed development processes, such as seed oilbody biogenesis (GO:0010344) and post-embryonic development (GO:0009791) (Figure 2c). These down-regulated DEGs were functionally involved in cell wall metabolism processes, such as the cell wall macromolecule metabolic process (GO:0044036) and the cell wall polysaccharide metabolic process (GO:0010383) (Figure 2c). These results indicate that DEGs at different developmental stages of oil-tea seeds are stage-specific.

### 2.3. Identification of Critical Candidate Genes Involved in Squalene Biosynthesis

Based on the squalene biosynthesis pathway and oil-tea genome [23], 42 putative genes involved in squalene biosynthesis were identified. These included 15 genes encoding enzymes (such as AACT, HMGR, MVK, and MVD) in the MVA pathway, 20 genes encoding enzymes (such as DXS, DXR, MCT, and HDS) in the MEP pathway, and 2, 3, and 2 genes encoding the enzymes IDI, FPS, and SS, respectively, in the IPP-related downstream pathway (Table S2).

**Figure 3:**
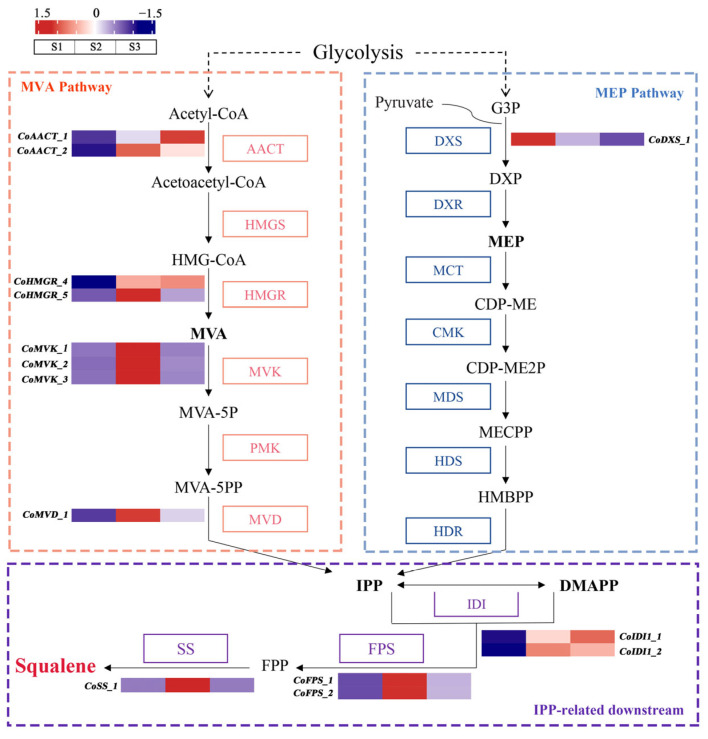
Expression pattern of DEGs involved in squalene biosynthesis across S1–S3. S1, S2, and S3 indicate three developmental stages of oil-tea seeds. The relative expression levels of genes were standardized RPKM values. Abbreviations: AACT, acetyl-CoA acetyltransferase; HMGS, 3-hydroxy-3-methylglutaryl-CoA synthase; HMG-CoA, 3-hydroxy-3-methylglutaryl coenzyme A; HMGR, 3-hydroxy-3-methylglutaryl-CoA reductase; MVA, mevalonate; MVK, mevalonate kinase; MVA-5P, mevalonate 5-phosphate; PMK, phosphomevalonate kinase; MVA-5PP, mevalonate 5-diphosphate; MVD, mevalonate diphosphate decarboxylase; G3P, D-glyceraldehyde-3-phosphate; DXS, 1-deoxy-D-xylulose-5-phosphate synthase; DXP, 1-deoxy-D-xylulose-5-phosphate; DXR, 1-deoxy-D-xylulose-5-phosphate reductoisomerase; MEP, 2-*C*-methyl-D-erythritol 4-phosphate; MCT, 2-*C*-methyl-D-erythritol 4-phosphate cytidylyltransferase; CDP-ME, 4-(cytidine 5’-diphospho)-2-*C*-methyl-D-erythritol; CMK, 4-(cytidine 5’-diphospho)-2-*C*-methyl-D-erythritol kinase; CDP-ME2P, 2-phospho-4-(cytidine 5’-diphospho)-2-*C*-methyl-D-erythritol; MDS, 2-*C*-methyl-D-erythritol 2, 4-cyclodiphosphate synthase; MECPP, 2-*C*-methyl-D-erythritol 2, 4-cyclodiphosphate; HDS, 4-hydroxy-3-methylbut-2-enyl-diphosphate synthase; HMBPP, 4-hydroxy-3-methylbut-2-enyl-diphosphate; HDR, 4-hydroxy-3-methylbut-2-enyl diphosphate reductase; IPP, isopentenyl diphosphate; DMAPP, dimethylallyl diphosphate; IDI, isopentenyl diphosphate isomerase; FPS, farnesyl diphosphate synthase; FPP, farnesyl diphosphate; and SS, squalene synthase.

Of the 42 genes involved in squalene biosynthesis, 13 genes exhibited a significant up-regulation at the S2 stage compared with the S1 stage, which is associated with squalene accumulation (Figure 3). Eight genes such as *CoAACT_1*, *CoAACT_2*, *CoHMGR_4*, *CoHMGR_5*, *CoMVK_1, CoMVK_2*, *CoMVK_3,* and *CoMVD_1* in the MVA pathway demonstrated up-regulated expression at the S2 stage compared with the S1 stage. Notably, *CoHMGR_4* and *CoAACT_2* exhibited 158.09-fold and 11.12-fold higher expression at the S2 stage compared to the S1 stage, respectively (Table S2). Within the MEP pathway, only *CoDXS_1* was significantly down-regulated at the S2 stage, whereas other genes did not show significant expression differences between the S1 and S2 stages (Figure 3). Similarly, *CoIDI_1*, *CoIDI_2*, *CoFPS_1*, *CoFPS_2*, and *CoSS_1* within the IPP-related downstream pathway also exhibited a significant up-regulated expression. Particularly, *CoFPS_1* and *CoFPS_2* demonstrated 13.71-fold and 14.09-fold higher expression at the S2 stage compared to the S1 stage, respectively (Table S2). These results indicated that the precursor IPP obtained via the MVA pathway is dominant rather than the MEP pathway. The 13 high-expressed genes at the S2 stage might be critical in mediating squalene accumulation as key enzymes in the seed development of oil-tea. Notably, *CoHMGR_4* exhibited significantly higher expression levels, with an average RPKM value of 256.10, than other squalene biosynthesis genes at the S2 stage, suggesting that it might play a vital role in squalene biosynthesis of developing oil-tea seeds. Furthermore, among the 13 candidate squalene synthase genes, four genes in the MVA pathway (*CoAACT_1*, *CoAACT_2*, *CoHMGR_4*, and *CoHMGR_5*) were significantly up-regulated at the S3 stage compared with the S1 stage, while *CoIDI_1* and *CoIDI_2* in the IPP-related downstream pathway were similarly up-regulated at the S3 stage (Figure 3). However, other genes, such as *CoMVK_1*, *CoFPS_1*, *CoFPS_2,* and *CoSS_1,* showed either no significant change or significant down-regulation at the S3 stage.

**Figure 4:**
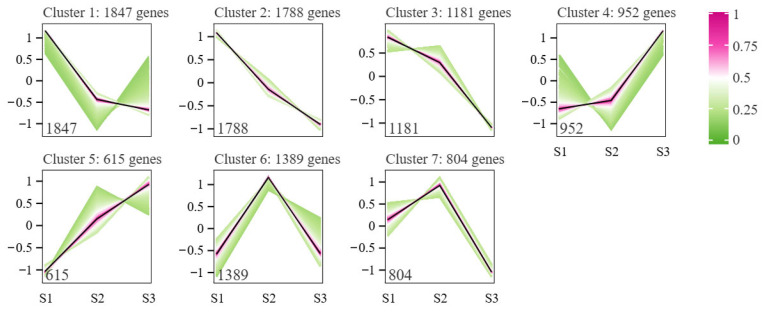
Mfuzz clustering analysis of DEGs based on gene expression patterns. The x-axis represents the three developmental stages (S1, S2, and S3) of oil-tea seeds. The y-axis indicates the standardized RPKM values. Numbers in the figure indicate the number of DEGs in the corresponding cluster.

To identify potential transcription factors that might regulate squalene biosynthesis in developing oil-tea seeds, we performed a co-expression Mfuzz clustering analysis for all identified DEGs, resulting in seven distinct clusters (Figure 4). Based on an assumption that the expression of genes involved in squalene biosynthesis might be associated with squalene accumulation with seed development, we paid more attention to the up-regulation of genes at the S2 stage compared to the S1 stage. Thus, we focused on the expression patterns in Clusters 5–7 and identified 13 key enzyme genes involved in squalene biosynthesis including *CoAACT_1*, *CoAACT_2*, *CoHMGR_4*, *CoHMGR_5*, *CoMVD_1*, *CoMVK_1*, *CoMVK_2*, *CoMVK_3*, *CoIDI_1*, *CoIDI_2*, *CoFPS_1*, *CoFPS_2*, and *CoSS_1*. Moreover, 258 TFs were identified in Clusters 5–7, including ERF (35 TFs), WRKY (29 TFs), bHLH (24 TFs), and MYB (23 TFs) as the most abundant. These 258 TFs exhibited similar expression patterns to the candidate key enzyme genes of squalene biosynthesis.

**Figure 5:**
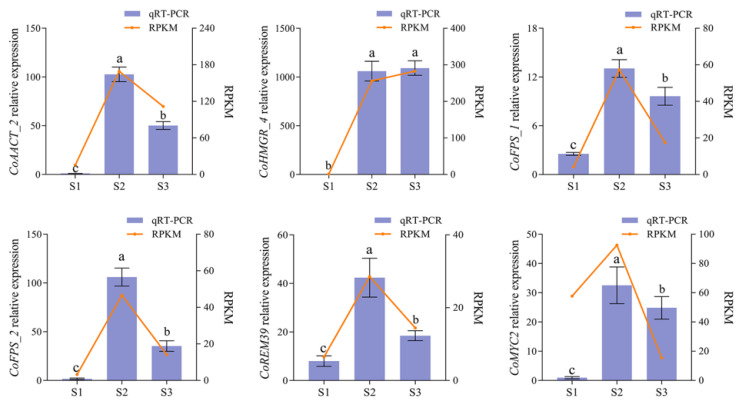
Quantitative RT-PCR validation of the six candidate genes involved in squalene biosynthesis. The x-axis represents the three developmental stages (S1, S2, and S3) of oil-tea seeds. Relative expression of qRT-PCR (the mean ± SD) calculated using *CoGAPDH* as the reference gene is shown in the left y-axis. RNA-seq expression of the gene (RPKM) is shown in the right y-axis. The letters above the error bars indicate statistical differences determined by one-way ANOVA (*p* < 0.05).

To experimentally validate the expression patterns of the identified DEGs encoding critical key enzymes and transcription factors involved in squalene biosynthesis that were obtained from the transcriptomic data, we performed qRT-PCR analysis for the six identified genes exhibiting higher expression levels at the S2 stage and greater fold change in up-regulation compared to the S1 stage, including four enzyme genes (*CoAACT_2*, *CoHMGR_4*, *CoFPS_1,* and *CoFPS_2*) and two TFs (CoMYC2 and CoREM39) in Clusters 5–7. The results revealed that the relative expression of the six tested genes exhibited a low expression in the S1 stage, a higher expression in the S2 stage, and a changing expression in the S3 stage (Figure 5). The qRT-PCR expression patterns were largely consistent with that from transcriptomic data. These results suggest that the expression patterns of genes involved in squalene biosynthesis were, indeed, associated with squalene accumulation in oil-tea seed development.

### 2.4. Validation of the Regulation of Squalene Biosynthesis Genes by Transcription Factor CoMYC2

**Figure 6:**
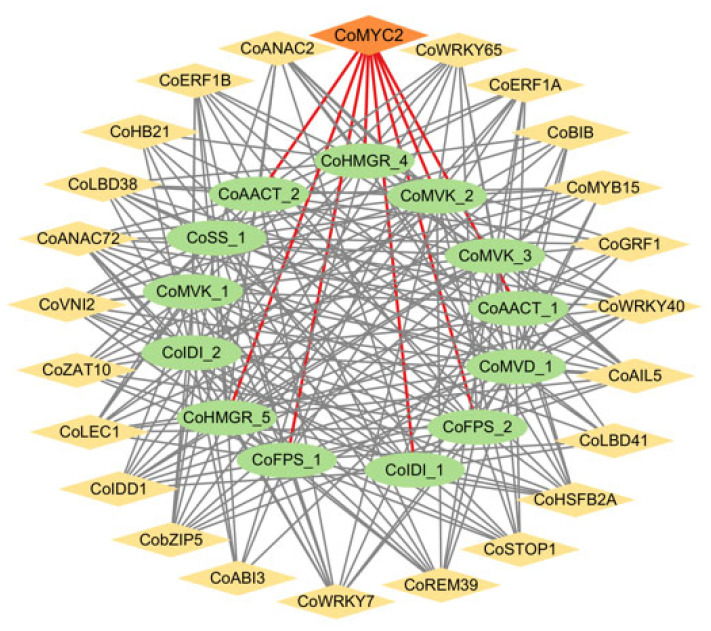
Co-expression network of 24 TFs and 13 squalene biosynthesis genes. Yellow and orange labels indicate transcription factors. Green labels indicate squalene biosynthesis genes. The red line highlights the potential regulatory relationships between CoMYC2 and squalene biosynthetic genes.

To identify candidate transcription factors potentially regulating squalene biosynthesis, a co-expression network was constructed between 13 key enzyme genes and 258 potential transcription factors using GENIE3 software. In total, 24 transcription factors (such as CoMYC2, CoREM39, CobZIP5, CoERF, and CoWRKY) showed high co-expression with the 13 genes encoding key enzymes, forming a potential co-expression network (Figure 6).

**Figure 7:**
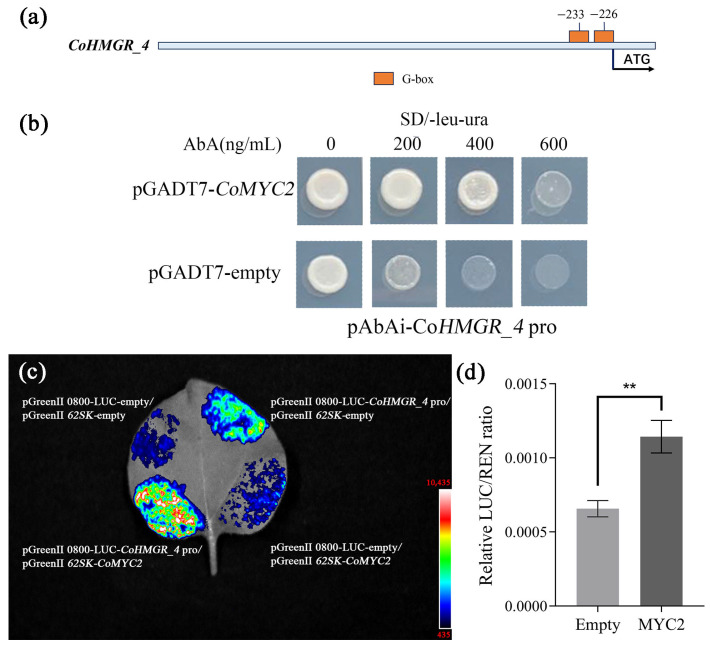
CoMYC2 directly binds to the promoters of *CoHMGR_4*. (**a**) The potential CoMYC2 binding sites (G-box) of the *CoHMGR_4* promoter. (**b**) Identification of the interaction between CoMYC2 and the *CoHMGR_4* promoter by yeast one-hybrid assay. Yeast cells were grown in an SD/-leu-ura selection medium containing different concentrations of aureobasidin A (AbA). The amounts of 0, 200, 400, and 600 indicate the concentration of AbA. The empty vector of pGADT7 was used as a negative control. (**c**) Identification of the interaction between CoMYC2 and the *CoHMGR_4* promoter by dual-luciferase activity assay. The pseudocolor bar indicates the fluorescence intensity of the LUC reporter gene. (**d**) The results of the relative LUC/REN ratio. Empty (pGreenII 0800-LUC-*CoHMGR_4* pro + pGreenII-62SK-empty) represents the negative control. MYC2 (pGreenII 0800-LUC-*CoHMGR_4* pro + pGreenII-62SK-*CoMYC2*) represents the experimental group. Bars represent the mean ± standard error of triplicate samples. Asterisk Indicates statistically significant differences determined by unpaired *t*-test (** *p* < 0.01).

Since a previous study revealed that squalene biosynthesis is induced by the jasmonic acid (JA) signal and MYC2 is mediated by the JA signal [24,25], we focused on a JA-responsive transcription factor, CoMYC2. We also noted that a DEG *CoHMGR_4* encoding the key enzyme HMGR co-expressed with CoMYC2 (Figure 6). To verify the functional regulation of CoMYC2 in squalene biosynthesis with oil-tea seed development, we thus selected *CoHMGR_4* and CoMYC2 to conduct the functional test. Firstly, we predicted cis-acting elements within the 2 kb promoter region upstream of the *CoHMGR_4* transcription start site on the genome. The diverse cis-acting regulatory elements were identified, including G-box, W-box, and the ethylene-responsive element (Figure S3). In particular, we noted the two G-boxes at the -226 and -233 positions (Figure 7a), which potentially bind the transcription factor CoMYC2. To verify whether CoMYC2 directly binds to the *CoHMGR_4* promoter and regulates its expression, yeast one-hybrid vectors (pAbAi-*CoHMGR_4* pro and pDADT7-*CoMYC2*) were constructed. The results of yeast one-hybrid assay showed that yeast strains carrying the vector (pDADT7-*CoMYC2*) were able to grow on selective media in the presence of 200 and 400 ng/mL AbA, whereas the yeast strain containing the pDADT7-empty vector exhibited only faint growth on selective media (Figure 7b). This suggested that CoMYC2 acted as a regulator to activate the expression of *CoHMGR_4*. Further, we performed a dual-luciferase assay in *Nicotiana benthamiana* leaves. Dual-luciferase assay vectors, pGreenII 0800-LUC-*CoHMGR_4* pro and pGreenII-62SK-*CoMYC,2* were constructed and introduced into *N. benthamiana* leaves, which were compared with the control transformed with empty vectors without *CoHMGR_4* pro or *CoMYC2*. The results demonstrated that CoMYC2 activated the expression of *CoHMGR_4* by directly binding to the promoter of *CoHMGR_4* (Figure 7c). Based on the expression ratio of firefly LUC to Renilla luciferase (LUC/REN), the LUC/REN ratio exhibited a significant change (Figure 7d). Taken together, we validated that CoMYC2 could directly regulate the expression of *CoHMGR_4*, suggesting that CoMYC2 might be a critical regulator in mediating squalene biosynthesis with oil-tea seed development.

## 3. Discussion

Oil-tea seed oils have largely been considered valuable edible oils depending on the relatively high content of squalene because of the active functions of squalene in maintaining human health. Squalene accumulation often occurs in diverse plants, and the mechanism of squalene biosynthesis and its molecular regulation remain largely uncertain in a given plant. It is essential to dissect the molecular basis of squalene accumulation in developing seeds for breeding or creating oilseed crops with a high content of squalene. In this study, we explored the molecular basis of squalene biosynthesis in developing oil-tea seeds and identified candidate genes involved in squalene biosynthesis, providing insights into understanding the mechanism of squalene biosynthesis in plant seeds.

The fast accumulation of squalene occurred in the middle stage of oil-tea seed development, which is consistent with squalene accumulation in developing seeds of *Camellia chekiangoleosa* [26]. Similarly, the fast accumulation of squalene mainly occurred from the middle stage to late stages of seed development in *Chaenomeles japonica* seeds [27]. However, the fast accumulation of squalene mainly occurred at the early stage of seed development in *Linum usitatissimum* [28]. These investigations suggest that patterns of squalene accumulation with seed development might vary in different plants.

Generally, there are two pathways for squalene biosynthesis in plants, i.e., dependent on the MVA pathway or MEP pathway. Based on our transcriptomic analyses, we found that eight genes encoding key enzymes in the MVA pathway were up-regulated at the accumulation stages of squalene, while none gene encoding squalene biosynthesis enzymes were up-regulated at the fast squalene accumulation stage in the MEP pathway. In other words, the precursor IPP for squalene biosynthesis downstream was predominantly derived from the MVA pathway (Figure 3). Thus, it is reasonable to assume that the MVA pathway is dominant in developing oil-tea seeds. Similarly, the MVA pathway is dominant in developing seeds of *M. sativa* [15], *C. sinensis* [29], and *H. brasiliensis* [30]. Studies have demonstrated that the MVA pathway is specifically responsible for the biosynthesis of triterpenoids, certain sesquiterpenes, and the ubiquinone side chain, while the MEP pathway often provides precursors for the downstream biosynthesis of monoterpenes, certain sesquiterpenes, diterpenes, carotenoids, the side chains of chlorophylls, and plastoquinone [11,14].

Based on the comparative transcriptomic analyses, we identified several candidate genes encoding key enzymes, such as *CoHMGR_4, CoAACT_2*, *CoFPS_1* and *CoFPS_2,* that might be crucial to control squalene biosynthesis in developing oil-tea seeds. The enzyme AACT is critical in catalyzing acetyl-CoA into acetoacetyl-CoA as the first step in the MVA pathway and restricts the biosynthesis of squalene and triterpenoid in *Taraxacum brevicorniculatum* [31]. Overexpression of *EkAACT* (cloned from *Euphorbia kansui*) in Arabidopsis resulted in a significant increase in squalene and triterpenoid accumulation [32]. Overexpression of *ZjFPS* significantly increased the squalene and triterpenoid contents in developing seeds of *Ziziphus jujuba,* while silencing of *ZjFPS* significantly decreased the contents [33]. The activity of the AtFPS enzyme was tightly correlated with the squalene and sitosterol contents in Arabidopsis seeds [34]. Studies have revealed that HMGR functioned as the rate-limiting enzyme in the MVA pathway [14,35]. Overexpressing *PgHMGR* in adventitious roots increased sterols and triterpenoid saponin contents in *P. ginseng* [36]. Overexpression of *HbHMGR* significantly enhanced the phytosterol and latex contents in rubber trees [37,38]. Taken together, although the MVA pathway of squalene biosynthesis might be conserved, key enzymes such as HMGR, AACT, and FPS are essential. However, their functions in controlling squalene biosynthesis or the rate-limiting of squalene biosynthesis might vary in different plants. Here, a couple of genes encoding several key enzymes involved in squalene biosynthesis were identified in developing seeds of oil-tea. Functional verification of these candidate genes in controlling squalene biosynthesis with oil-tea seed development deserves further research.

The TF regulation of squalene biosynthesis usually determines whether squalene biosynthesis is activated and the content of squalene accumulation. Several studies have found that bZIP members such as MtbZIP17 and MtbZIP60 are involved in regulating the biosynthesis of triterpene saponins by activating the expression of key enzyme genes such as *HMGR* in *M. truncatula* [39]. Similarly, EsbZIP1, EsbZIP2, EsbZIP4, and EsbZIP5 are critical in regulating the biosynthesis of triterpene saponins by inhibiting the expression of key enzyme genes including *FPS* and SS in *Eleutherococcus senticosus* [40]. The ERF members such as GAME9, JRE4, and ERF1 have been identified to regulate sterol biosynthesis-related genes in Solanaceae plants and *Petunia hybrida* [41,42,43]. bHLH members such as TSAR1, TSAR2, and MYC2 were found to regulate squalene and triterpenoid biosynthesis in *M. truncatula* and Solanaceae plants [22,41]. The WRKY members such as WsWRKY1 from *Withania somnifera*, HbWRKY27 from *H. brasiliensis*, TgWRKY3 from *Torreya grandis*, and CoWRKY15 from *C. oleifera* cultivar ‘Huashuo’ have been demonstrated to activate the expression of *FPS* and *SS* in regulating squalene and sterol biosynthesis [19,44,45,46]. Here, 24 transcription factors potentially involved in squalene biosynthesis during the development of oil-tea seeds were identified, covering bHLH, bZIP, ERF, and WRKY families. Based on the co-expression network of identified TFs, these transcription factors function in regulating squalene biosynthesis by regulating multiple genes encoding key enzymes. There were studies showing that TaMYC2 and GpMYC2 were critical in enhancing squalene and triterpenoids by regulating the expression of *TaSS* and *GpFPS1* in *T. antungense* [24] and *Gynostemma pentaphyllum* [47], respectively. We validated the regulation of squalene biosynthesis by CoMYC2 activating *CoHMGR_4* using the yeast one-hybrid and dual-luciferase assays in this study. Taken together, MYC2 is most likely multi-functional in regulating squalene and triterpenoid biosynthesis via activating multiple key enzyme genes in plants.

## 4. Materials and Methods

### 4.1. Plant Materials

Eight clonal 12-year-old *C. oleifera* trees of the major Yunnan cultivar ‘Yunyoucha No. 4’ (YCO4) and one tree of ‘Yunyoucha No. 3’ (YCO3) were selected as experimental subjects. These trees were uniformly cultivated under water and fertilizer regimes in the Oil-tea Germplasm Base (24°07’ N, 105°07’ E), Guangnan Town, Yunnan Province, China, which is affiliated with the Yunnan Academy of Forestry and Grassland. The YCO4 cultivar flowers from late September to mid-November, with the peak flowering period occurring in early October. Therefore, artificial pollination of eight YCO4 clones was conducted from 2 to 8 October 2020, using YCO3 as the male parent. The flowers were emasculated and bagged at the bud stage to prevent stigma contamination by any pollen until they became receptive. Each flower was hand-pollinated with the mature pollen of YCO3 at least three times, prior to pistil wilting, and the pollinated flowers were labeled. According to the multi-year data of the phenotype, the seed of YCO4 takes 13 months to mature after pollination. The seeds remain dormant until the next June, and then they begin to expand in mid-June. Therefore, the fruits were collected starting from the early seed development stage at 236 DAPs (1 June 2021) to the seed mature stage at 376 DAPs (20 October 2021), covering eight sampling time points (236, 256, 276, 296, 316, 336, 356, and 376 DAPs). For each sampling time point, 50–200 fruits were randomly collected from eight YCO4 clones and used as experimental materials in this study. From each sampling point, seeds from three fruits were immediately frozen in liquid nitrogen and stored at −80 °C for RNA-seq and qRT-PCR analyses, while the remaining seeds were used for measurements of seed dry weight, oil weight, and squalene content.

### 4.2. Measurement of Seed Dry Weight, Oil Weight, and Squalene Content

After shelling, the fresh seeds from oil-tea fruits were randomly assigned to three biological replicates and dried in an oven at 45 °C to a constant weight. The total seed dry weight of each biological replicate was measured using a balance, and the single seed dry weight was calculated by dividing the total weight by the number of seeds. For each biological replicate, the dried seeds were ground into powder. Approximately 2 g of the powder was weighed in a filter paper bag, and the oil content was determined using a Sox406 Soxtec apparatus (Hanon, Jinan, China), following the method of Gong et al. [48]. To determine squalene content, approximately 0.25 g of the extracted oil was purified through a silica gel column (10 mm × 250 mm) using n-hexane as the eluent and subsequently analyzed by Gas Chromatography–Mass Spectrometry (GC-MS) using a Trace 1310-ISQ 7000 system (Thermo, Waltham, MA, USA)) equipped with an HP-5MS column (Agilent Technologies, Santa Clara, CA, USA). In split injection mode with a 10:1 split ratio, 1 μL of the sample was injected into the column with helium as the carrier gas (flow rate: 1 mL/min). The column temperature program was initiated at 180 °C for 2 min, increased at a rate of 15 °C/min to 300 °C, and held at this temperature for 5 min. The temperatures of the injector, quadrupoles, and ion source were set to 280 °C, 150 °C, and 230 °C, respectively. The MS was operated in electron ionization (EI) mode, and ion analysis was performed in single-ion monitoring (SIM) scan mode. The ions at *m*/*z* 81, 95, and 137 were used for confirmation, while the ion at *m*/*z* 69 was used for quantification. The total GC-MS run time was 11.5 min, with a 3 min solvent delay. The squalene standard concentrations were set at 10, 20, 40, 90, and 180 μg/mL.

Oil weight of single seed = oil content/weight of powder × single seed dry weight; squalene content of single seed = squalene content/weight of oil × oil weight of single seed.

### 4.3. Construction of Illumina RNA-Seq Library for Sequencing and Functional Annotation

Total RNA from seeds at different stages of squalene accumulation (236 DAPs, 256 DAPs, and 316 DAPs) was extracted using the RNAprep Pure Plant Kit (TransGen Biotech, Beijing, China), following the manufacturer’s protocols. RNA concentration and integrity were evaluated using a NanoDrop 2000 spectrophotometer (Thermo, Waltham, MA, USA) and an Agilent 2100 Bioanalyzer (Agilent Technologies, Santa Clara, CA, USA), respectively. The cDNA libraries were constructed and sequenced on the Illumina NovaSeq 6000 platform at OE Biotech Company (Shanghai, China). Three independent transcriptome databases for sampling at the same accumulated stage were constructed. Trimmomatic (version 0.36) [49] was used to remove adapters, poly-N sequences, and low-quality reads. The high-quality clean reads were mapped to the *C. oleifera* reference genome [23] using HISAT2 (version 2.2.1) software [50]. The functional annotation of genes was carried out by BLASTx (version 2.13.0) search with the Kyoto Encyclopedia of Genes and Genomes (KEGG) (Release 105.0) and the Gene Ontology (GO) (version 2023-01-01) public database. Gene abundances were calculated and normalized to reads per kilobase of transcript per million mapped reads (RPKM) using TBtools (version 2.309) software [51]. Differentially expressed genes (DEGs) were identified using |fold-change| ≥ 2 and *p*-value ≤ 0.05 using DEseq2 (version 1.36) [52].

### 4.4. Identification of Squalene Biosynthesis Genes and Transcription Factors

The squalene biosynthesis genes were identified based on homologous genes of *A. thaliana* [14] available at https://www.arabidopsis.org/ (accessed on 12 January 2023) using BLAST (version 2.13.0) with TBtools software with *E* value cutoff 1 × 10^−5^. The domains of the identified genes were examined using SMART (http://smart.embl-heidelberg.de/; accessed on 22 March 2023) and Pfam (http://pfam-legacy.xfam.org/; accessed on 22 March 2023). Transcription factors in the *C. oleifera* reference genome were identified using PlantTFDB (http://planttfdb.gao-lab.org/index.php; accessed on 10 May 2023).

### 4.5. Quantitative Real-Time PCR (qRT-PCR)

The cDNA was synthesized from total RNA using the TransScript^®^ All-in-One First-Strand cDNA Synthesis SuperMix for qRT-PCR kit (TransGen Biotech, Beijing, China). Primers were designed with the NCBI Primer-BLAST tool (https://www.ncbi.nlm.nih.gov/tools/primer-blast/; accessed on 10 October 2023) and are listed in Table S3. qRT-PCR amplifications were performed using the PerfectStart^®^ Green qPCR SuperMix kit (TransGen Biotech, Beijing, China) with the following cycling conditions: 95 °C for 30 s, followed by 40 cycles of 95 °C for 5 s, 59 °C for 20 s, and 72 °C for 12 s. Each gene was tested with three biological replicates and each biological replicate had three technical replicates. *CoGAPDH* was used as the reference gene [48], and relative expression levels were calculated using the 2^−ΔΔCt^ method [53].

### 4.6. Construction of Co-Expression Network and Cis-Acting Element Analysis

Meta-Fuzzy Clustering (Mfuzz) analysis [54] was performed using OmicShare tools (http://www.omicshare.com/tools; accessed on 15 May 2023) to analyze gene expression patterns based on standardized RPKM values. The co-expression network between TFs and candidate squalene biosynthesis genes was constructed using the R package GENIE3 (version 1.30.0) [55]. TFs with RPKM values less than 15 at the S2 stage, weight values less than 0.03, and node degrees less than 6 in the network were filtered out. The co-expression network was visualized via Cytoscape (version 3.8.2) [56]. The promoter sequences of *CoHMGR_4* (2 kb upstream of transcription start site) were retrieved from the reference genome using TBtools software. The potential cis-acting elements of the *CoHMGR_4* promoter were predicted by PLACE (https://www.dna.affrc.go.jp/PLACE/?action=newplace; accessed on 9 October 2023) and JASPAR (https://jaspar.elixir.no/; accessed on 9 October 2023).

### 4.7. Yeast One-Hybrid Assays

Promoter fragments (150 bp) containing the G-boxes of *CoHMGR_4* and the full-length coding sequence (CDS) of CoMYC2 were cloned via PCR using specific primers (Table S4). The amplified sequences were validated by Sanger sequencing. The *CoHMGR_4* promoter fragments were cloned into the *SacI* and *SalI* sites of the pAbAi vector as the bait, and the full-length CDS of *CoMYC2* was fused into the *NdeI* and *BamHI* sites of the pGADT7 vector to construct the prey. The recombinant vectors were then transformed into the yeast Y1H Gold strain via the homologous recombination and selected on SD/−leu−ura medium containing different concentrations of aureobasidin A (AbA). Protein–DNA interactions were assessed based on the growth ability of the yeast cells.

### 4.8. Dual-Luciferase Assays

The full-length CDS of *CoMYC2* was cloned into the *BsaI* site of the pGreenII-62SK vector as the effector. Promoter fragments of *CoHMGR_4* (2 kb upstream of transcription start site) were cloned as described and fused in the *BsaI* sites of the pGreenII 0800-LUC vector to construct the reporter. The sequence-specific primers are listed in Table S5. The empty pGreenII-62SK vector (without *CoMYC2*) and the empty pGreenII 0800-LUC vector (without the *CoHMGR_4* promoter) were used as negative controls. The constructed effector and reporter vectors were co-transformed into *N. benthamiana* leaves following the previously reported method [57]. The transformed plants were grown under weak light for 2 days and then divided into two groups. For one group, the leaves were placed in the dark for 10 min after being sprayed with D-luciferin potassium salt, and fluorescence signals were detected and imaged using the Tanon 5200 chemiluminescence analyzer (Tanon, Shanghai, China). For the other group, leaf injection sites were excised, and luciferase activity was measured using the VICTOR^®^ Nivo™ microplate reader (PerkinElmer, Waltham, MA, USA), following the Dual Luciferase Reporter Assay Kit (Vazyme, Nanjing, China) protocol. The LUC/REN ratio was calculated as the final transcriptional activity, with three biological replicates.

## 5. Conclusions

Through comparative transcriptome analysis of oil-tea seeds at the early, fast biosynthesis, and late stages of squalene accumulation, our study revealed that the precursor IPP of squalene biosynthesis was predominantly derived from the MVA pathway in oil-tea seed development. A total of 13 candidate key enzyme genes involved in squalene biosynthesis were identified, including eight genes in the MVA pathway (such as *CoHMGR_4* and *CoAACT_2*) and five genes in the IPP-related downstream pathway (such as *CoFPS_1* and *CoFPS_2*). Notably, *CoHMGR_4* exhibited significant up-regulation at the fast biosynthesis stage, suggesting its crucial role in squalene biosynthesis. Furthermore, diverse transcription factors, including members of the bHLH, ERF, and WRKY families, were identified as potentially involved in the regulation of squalene biosynthesis. Among these, the transcription factor CoMYC2 might be a critical regulator of squalene biosynthesis during oil-tea seed development by activating the expression of the key enzyme gene *CoHMGR_4*. These findings provide insights into the molecular basis of squalene biosynthesis in developing oil-tea seeds.

## Data Availability

All data are contained within the article and the Supplementary Materials.

## Supplementary Materials

The following supporting information can be downloaded at https://www.mdpi.com/article/10.3390/ijms26125465/s1.
