# Supplementary material for: Comparative Transcriptomic Analysis Reveals the Potential Molecular Mechanism Underlying Squalene Biosynthesis in Developing Seeds of Oil-Tea (Camellia oleifera)
Source: Int J Mol Sci. 2025 Jun 7;26(12):5465. doi: 10.3390/ijms26125465 (PMC12193504; doi:10.3390/ijms26125465)
Supplement: Supplementary file 1 [file ijms-26-05465-s001.zip › Supplementary Materials Figures.pdf]

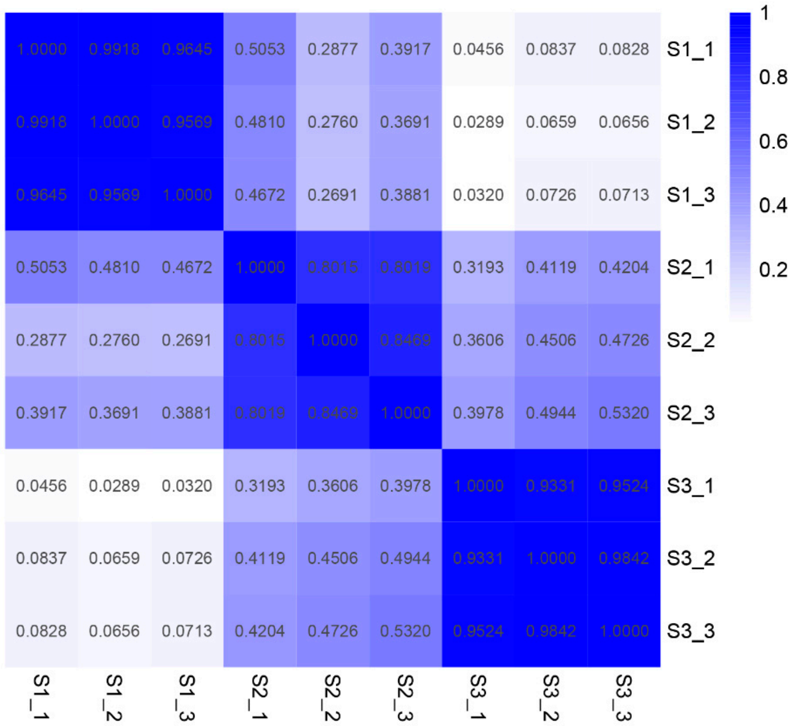

Figure S1. Pearson correlation coefficient of transcriptome dataset.

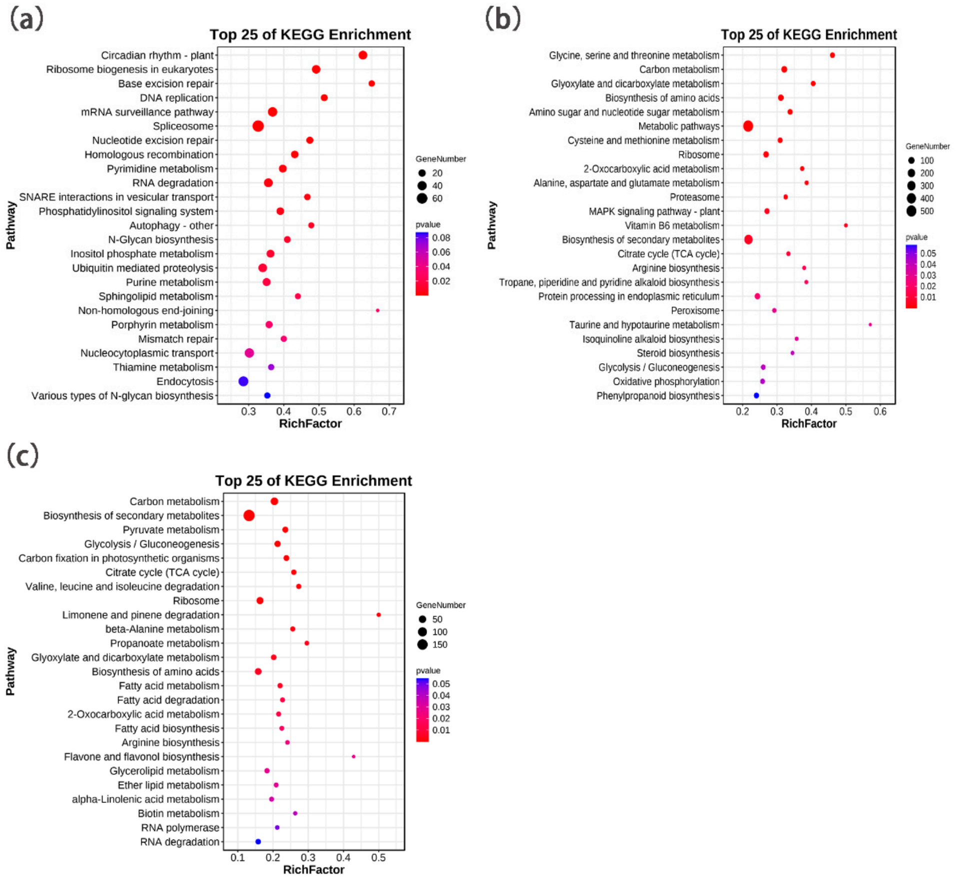

Figure S2. KEGG enrichment analysis of highly expressed genes at S1(a), S2(b) and S3(c) stages of oil-tea seed development.

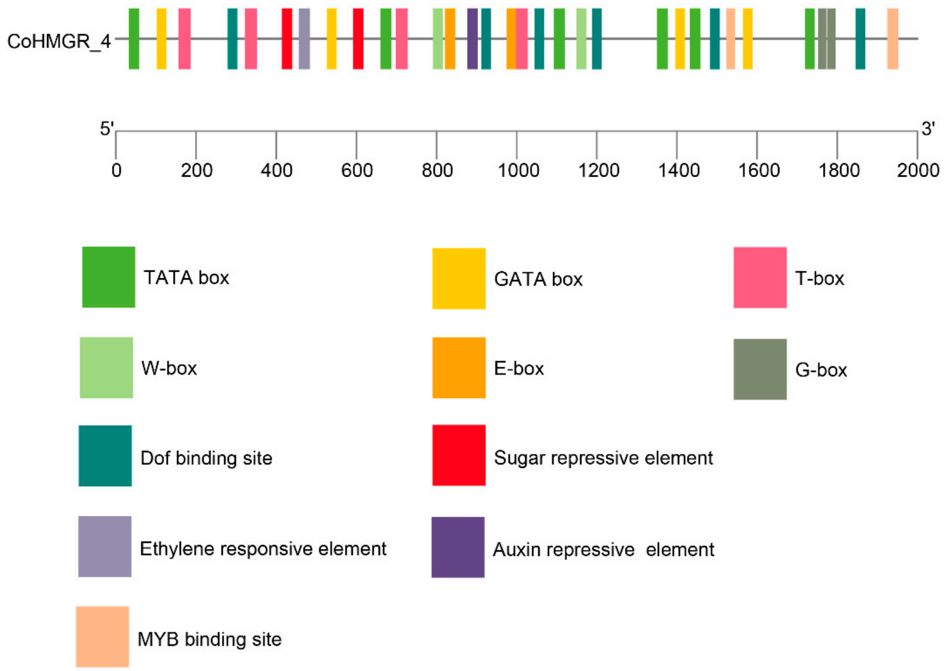

Figure S3. Cis-acting element analysis of *CoHMGR_4* promoter.
